# Glenohumeral joint septic arthritis and osteomyelitis caused by *Moraxella catarrhalis* after arthroscopic rotator cuff repair: case report and literature review

**DOI:** 10.5194/jbji-9-225-2024

**Published:** 2024-10-18

**Authors:** Yong-Beom Kim, Jinjae Kim, Min Gon Song, Tae Hyong Kim, Tae-Yoon Choi, Gi-Won Seo

**Affiliations:** 1 Department of Orthopedic Surgery, Soonchunhyang University Seoul Hospital, Seoul 04401, South Korea; 2 Division of Infectious Diseases, Department of Internal Medicine, Soonchunhyang University Seoul Hospital, Seoul 04401, South Korea; 3 Department of Laboratory Medicine, Soonchunhyang University Seoul Hospital, Seoul 04401, South Korea

## Abstract

*Moraxella catarrhalis* commonly colonizes the upper respiratory tract of humans, but infection caused by *M. catarrhalis* after orthopedic surgery is rare. Here, we report the first case of septic arthritis of the shoulder caused by an *M. catarrhalis* infection and outline the diagnosis and treatment steps as well as differences compared with other cases.

## 1 Introduction

*Moraxella catarrhalis* is an aerobic, nonmotile, Gram-negative diplococcus that commonly colonizes the upper respiratory tract of humans. It is uncommonly associated with invasive disease unless the patient is in an immunocompromised state. Common clinical manifestation are limited to otitis media and respiratory tract infections, such as sinusitis, bronchitis, and pneumonia (Verduin et al., 2002). Septic arthritis, vertebral osteomyelitis, and diskitis caused by *M. catarrhalis* without concomitant bacteremia have rarely been reported (Brunckhorst et al., 2020; Craig and Wehrle, 1983; Evangelista et al., 2017; Izraeli et al., 1989; Leonardou et al., 2005; Melendez and Johnson, 1991; Olivieri et al., 2004; Prallet et al., 1991; Minoza et al., 2015). In septic arthritis, prompt diagnosis and treatment with antibiotics and surgery are crucial to prevent further joint damage and ensure a good prognosis (Shirtliff and Mader, 2002). Septic arthritis of the glenohumeral joint caused by *M. catarrhalis* has not yet been reported. We present the case of a patient who developed septic arthritis of the right glenohumeral joint caused by *M. catarrhalis* after arthroscopic rotator cuff repair. In addition, we review the literature in the field of orthopedics for case reports of septic arthritis caused by *M. catarrhalis*.

## 2 Case presentation

The patient provided written informed consent for the publication of this report and the accompanying images.

### 2.1 Clinical presentation

A 71-year-old patient with hypertension and hyperlipidemia presented with a 2-year history of right shoulder pain. The patient was diagnosed with a rotator cuff tear and impingement syndrome at another hospital and received acupuncture and prolotherapy until October 2022 at a local clinic. Their symptoms worsened after they had a fall on 24 December 2022. The patient received local steroid injections in early January 2023, but the symptoms did not improve. On 12 January 2023, the patient underwent arthroscopic rotator cuff repair and acromioplasty at another hospital. On postoperative day (POD) 5, they developed a mild fever and swelling, a burning sensation, and redness of the right shoulder. The patient was closely monitored via regular follow-ups, but no discharge was noted. Laboratory parameters – such as white blood cell (WBC) count (10 000 $c\;µL^{-1}$ vs. normal range of 4000–10 000 $c\;µL^{-1}$ ) and C-reactive protein (CRP) level (9.7 $\mathrm{mg}\;{\mathrm{dL}}^{-1}$ vs. normal range of 0.0–0.5 $\mathrm{mg}\;{\mathrm{dL}}^{-1}$ ) – were elevated. Cefazolin administration was started on POD 5. Despite receiving cefazolin until POD 7, there was no improvement in the symptoms. Therefore, the patient was transferred to our hospital. At admission, the patient had no fever, and laboratory analysis showed that their WBC count was 8500 $c\;µL^{-1}$ and CRP level was 7.28 $\mathrm{mg}\;{\mathrm{dL}}^{-1}$ .

**Figure 1:**
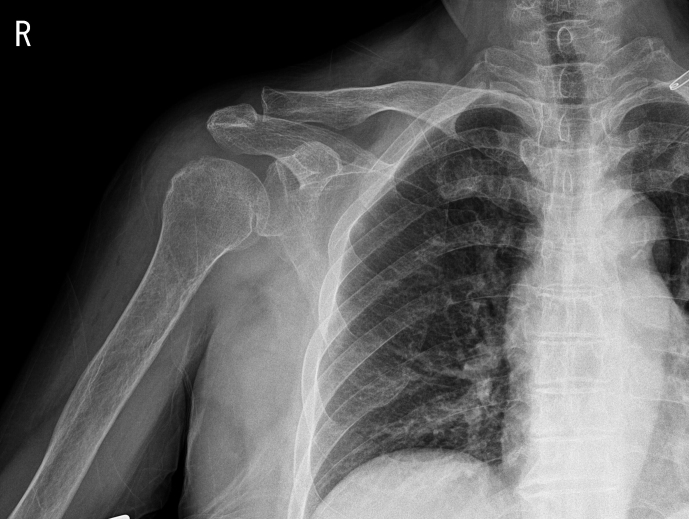
Plain radiograph showing osteoarthritic changes in the right acromioclavicular joint and soft-tissue swelling around the right shoulder.

**Figure 2:**
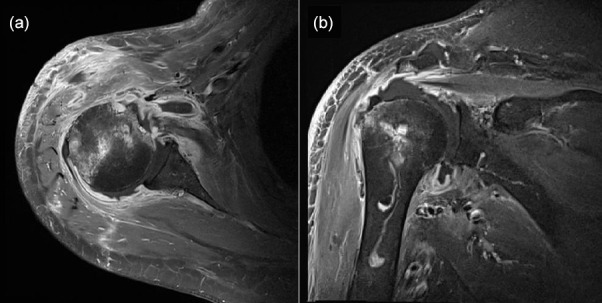
Enhanced magnetic resonance images showing effusion in the right glenohumeral joint with synovial hypertrophy and combined diffuse heterogeneous enhancement along the right glenohumeral joint capsule. Additionally, an ill-defined bone marrow signal intensity change is observed in the right humeral head. Panel **(a)** presents a T1-weighted, contrast-enhanced fat suppression axial-view image, whereas panel **(b)** shows a T1-weighted, contrast-enhanced fat suppression coronal-view image.

### 2.2 Imaging studies

Plain-film radiographs of the right shoulder showed osteoarthritic changes in the right acromioclavicular joint and soft-tissue swelling around the right shoulder (Fig. 1). Enhanced magnetic resonance imaging (MRI) showed effusion in the right glenohumeral joint with synovial hypertrophy and combined diffuse heterogeneous enhancement along the right glenohumeral joint capsule. In addition, an ill-defined change in the bone marrow signal intensity in the right humeral head was observed (Fig. 2). After full radiologic evaluation, multidisciplinary discussion was led by an orthopedic surgeon, an infectious disease specialist, a radiologist, and a clinical microbiologist. After team discussion, postoperative septic arthritis was suspected, and diagnostic and therapeutic arthroscopic surgery was scheduled. All previous antimicrobial agents were discontinued to improve the diagnostic yield of the causative pathogen in resection tissue culture. Given the know microbial epidemiology of postsurgical infection, the initial empirical antimicrobial agents after arthroscopic surgery should cover common Gram-positive skin flora as well as Gram-negative bacteria due to the patient's age.

**Figure 3:**
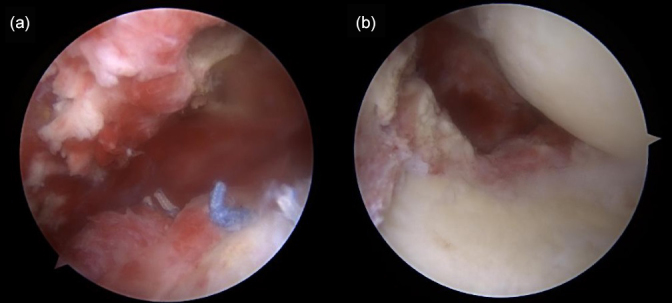
Arthroscopic findings showing **(a)** severe synovitis with pus-like fluid on the intra-articular side and **(b)** inflammatory granulation tissue and severe bursitis on the bursal side.

**Figure 4:**
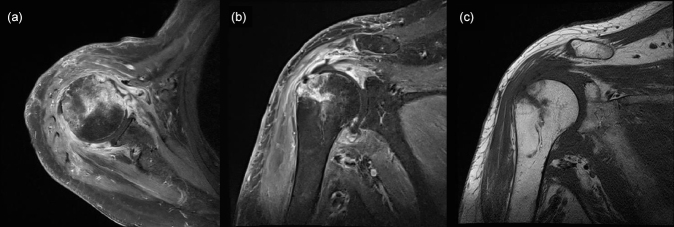
Magnetic resonance image obtained 3 weeks after surgery showing increased signal intensity in the cortex and medulla of the humeral head, while the same areas appear well-demarcated and darker in the non-enhanced image: **(a)** T1-weighted, contrast-enhanced fat suppression axial-view image; **(b)** T1-weighted, contrast-enhanced fat suppression coronal-view image; **(c)** T1-weighted, non-contrast-enhanced coronal-view image.

### 2.3 Treatment

Shoulder arthroscopy was performed on 20 January 2023 for irrigation, and specimens were obtained via debridement. The arthroscopic findings of the intra-articular side were severe synovitis with pus-like fluid, whereas those of the bursal side were inflammatory granulation tissue and severe bursitis with a well-maintained previously repaired rotator cuff (Fig. 3). Gram staining and pathology tests were performed on the specimen. The patient remained admitted postoperatively and was administered empirical intravenous vancomycin (2 $g\;d^{-1}$ ) and cefepime (6 $g\;d^{-1}$ ) while awaiting culture results until POD 5. Gram staining showed Gram-negative diplococcus, and subsequent tissue culture revealed *M. catarrhalis*. Based on pathological examination, the specimen turned out to be acute and chronic nonspecific inflammation with granulation tissue formation. Antimicrobial agents were de-escalated to vancomycin and ampicillin/sulbactam (3 $g\;d^{-1}$ ) until POD 20. The vancomycin targeting potential Gram-positive bacteria such as methicillin-resistant *Staphylococcus aureus* (MRSA) was not initially discontinued, even after confirmation of *M. catarrhalis* as the causative pathogen, because this bacteria was not expected to cause such surgical infection as commonly. MRI performed 3 weeks after surgery revealed that the bone marrow signal intensity of the humeral head had increased on a contrast-enhanced, T1-weighted image, and the marrow of the humeral head appeared darker and well-demarcated on a non-enhanced, T1-weighted image (Fig. 4). Concurrent osteomyelitis of humeral head was later diagnosed, and antimicrobial agents were administered for 6 weeks after surgery.

After 3 weeks of antibiotic administration, the patient had a systemic allergic rash; therefore, the antibiotics were changed to intravenous ceftriaxone alone (2 $g\;d^{-1}$ ). The medical treatment was then simplified to target only *M. catarrhalis* due to an unavoidable adverse drug event. Despite this, the systemic allergic rash persisted; therefore, the medication was changed to oral moxifloxacin (400 $\mathrm{mg}\;d^{-1}$ ). The rash seemed to improve for a while; however, 2 d after changing the antibiotic, the rash worsened again, so medication was changed to oral doxycycline (200 $\mathrm{mg}\;d^{-1}$ ). After changing antibiotics, allergy symptoms improved and treatment was maintained until 6 weeks after surgery.

Laboratory analysis performed after 6 weeks of antibiotic administration revealed normalized WBC counts and CRP levels of 7100 $c\;µL^{-1}$ and 0.17 $\mathrm{mg}\;{\mathrm{dL}}^{-1}$ , respectively. A total of 11 weeks after surgery, the patient reported a full range of motion in the right shoulder joint, with no significant limitations with respect to daily activities. Additionally, no notable symptoms were observed.

## 3 Discussion and conclusions

Septic arthritis is an unfortunate healthcare-associated complication of arthroscopy, with an overall estimated incidence of less than 1 % (Bauer et al., 2015). It is a potentially serious and debilitating condition that can occur when bacteria or other microorganisms infect a joint. Surgery, underlying joint disease, diabetes mellitus, presence of prosthesis, skin defect or infection, advanced age, and immunosuppressive medication are known risk factors of septic arthritis (García-Arias et al., 2011). Moreover, invasive procedures (similar to what the patient experienced in this case) could be a risk factor for septic arthritis; intra-articular steroid injections which can cause septic arthritis in 4 out of 10 000 cases (Geirsson et al., 2008) and various cases of septic arthritis associated with acupuncture have been reported (Woo et al., 2009).

The most common pathogens associated with shoulder infection after shoulder arthroscopy are *Cutibacterium acnes* and staphylococci (Bauer et al., 2015; Kumar and Thilak, 2016). Other pathogens that can cause shoulder infection include *Pseudomonas aeruginosa*, *Mycobacterium tuberculosis*, and *Actinomyces* (Aydin et al., 2014; Bauer et al., 2015; Khan et al., 2017; Pauzenberger et al., 2017). Here, we report the first case of septic arthritis of the shoulder caused by *M. catarrhalis* infection.

*Moraxella* is a genus of opportunistic pathogens that infect various body parts, including the respiratory tract, middle ear, and sinuses (Verduin et al., 2002). *Moraxella* is commonly associated with respiratory tract infections, often leading to conditions such as pneumonia. Typically, chest X-rays reveal a bronchopneumonia pattern, while computed tomography (CT) scans demonstrate bronchial wall thickening, bilateral distribution, and segmental patterns (Hirai et al., 2020). However, we present a case of *Moraxella* infection in which the patient did not exhibit respiratory symptoms; moreover, chest X-ray findings were unremarkable. It is known that *Moraxella* can cause meningitis and eye infections other than respiratory infections, but bone and joint infections have rarely been reported, and they have been described mostly in adults, particularly in those with underlying joint disease or immunodeficiency (Murphy and Parameswaran, 2009). Additionally, there are rare cases in which a pathogen has been reported as the causative bacteria of prosthetic joint infection. Two of the three prosthetic joint infection cases were immunocompromised patients (Evangelista et al., 2017; Minoza et al., 2015; Leonardou et al., 2005).

**Table 1:** Reported cases of infectious arthritis with *Moraxella catarrhalis*.

Year	Author	Age	Sex	Location	Prior	Comorbidity	Diagnosis	Antibiotics	Treatment
					operation			(period)	
1983	Craig andWehrle (1983)	23	Male	Right hip	n/a	n/a	Septic hiparthritis	Intravenous (IV) penicillin 3 500 000 U q4 (3 weeks)	Open debridement and open synovial biopsy
1989	Izraeli etal. (1989)	3 months	Female	Right knee, left hip	n/a	n/a	Septic knee and hip arthritis	IV cefuroxime IV gentamicin (2 weeks)	Open drainage of the hip joint
1991	Melendez and Johnson (1991)	41	Male	Left knee	n/a	IV heroin abuserfor 20 years, sexual relationswith a prostitute 4 d before admission	Septic kneearthritis	IV penicillin G 2 000 000 U q4 (12 d), 10 d laterrestart IV penicillin G 3 000 000 U q4 (7 d)	Arthrotomy and drainage of fluid
2004	Olivieri etal. (2004)	45	Male	Right knee	n/a	Undifferentiated spondarthritis treated withIV infliximab(5 mg kg^-1^ at 0, 2, 6, and 14 weeks)	Septic kneearthritis	Ciprofloxacin 1 g d^-1^ andteicoplanin 20 mg d^-1^ (2 weeks)	Joint drainage two times
2023	Rogers (2023)	15	Female	Right elbow	n/a	Systemic lupus erythematosus (SLE)	Septic elbowarthritis	IV ceftriaxone (10 d), oral amoxicillin/clavulanic acid(11 d)	Antibiotics

n/a: not applicable. q4: administration every 4 h.

We reviewed five published cases of septic arthritis on joints other than glenohumeral joints caused by *M. catarrhalis* (Table 1) (Craig and Wehrle, 1983; Izraeli et al., 1989; Melendez and Johnson, 1991; Olivieri et al., 2004; Rogers, 2023). One patient had an immunodeficiency as well as underlying joint disease, one other patient had an immunodeficiency with no joint disease, and three patients had no underlying joint disease or immunodeficiency. In three patients, surgical interventions such as arthrotomy and drainage, open drainage, and open debridement were performed along with antibiotic treatment; the remaining two patients only received antibiotic treatment and did not undergo surgery. Antibiotics, such as levofloxacin, clindamycin, ceftriaxone, and gentamicin, were administered for at least 2 weeks in five patients. Two patients reported by Craig and Wehrle (1983) and Melendez and Johnson (1991) received penicillin as their initial treatment.

The differences between our patient and those in previously published case reports are as follows: our patient had an underlying joint disease with no immunodeficiency. This is the first reported case of septic arthritis of the glenohumeral joint occurring after arthroscopic surgery. Our patient's septic arthritis progressed to osteomyelitis. After surgery, vancomycin and beta-lactams were administered to treat osteomyelitis; however, side effects (such as skin rash and itching) occurred, and the treatment was changed to cephalosporin antibiotics and doxycycline.

The clinical presentation of septic arthritis caused by *M. catarrhalis* is similar to that of other types of septic arthritis, with symptoms such as joint pain, swelling, stiffness, fever, and chills. However, diagnosis can be challenging because *M. catarrhalis* is not commonly associated with joint infection, and other causes of joint inflammation must be ruled out. Prompt and aggressive antibiotic therapy is necessary to control the infection and prevent further joint damage. *M. catarrhalis* is generally susceptible to beta-lactams, tetracycline, quinolones, and aminoglycosides. However, most *M. catarrhalis* isolates produce inducible beta-lactamase (Vaneechoutte et al., 2015). Surgical intervention such as early arthroscopic joint irrigation or debridement may be necessary in some cases. In conclusion, although *M. catarrhalis* is not a common cause of septic arthritis, it should be considered in the differential diagnosis of joint infection, particularly in adults with underlying joint diseases or immunodeficiency. Early diagnosis and appropriate antibiotic therapy are essential for achieving favorable outcomes and preventing long-term joint damage.

Infection caused by *M. catarrhalis* is rare after orthopedic surgery, and our study is the first to report septic arthritis of the glenohumeral joint. Although the clinical symptoms are similar to those of septic arthritis caused by commonly encountered bacteria, it is important to test for antibiotic susceptibility using culture. Joint damage should be minimized through appropriate antibiotic therapy and surgical intervention.

## Data Availability

The patient data generated and/or analyzed during the current study are presented in Figs. 1–4. Data from other existing publications discussed in this work are listed in Table 1.
